# Diagnosis and surgical treatment for isolated tricuspid libman-sacks endocarditis: a rare case report and literatures review

**DOI:** 10.1186/s13019-015-0302-1

**Published:** 2015-07-08

**Authors:** Zhixuan Bai, Jianglong Hou, Wenjun Ren, Yingqiang Guo

**Affiliations:** Department of Cardiovascular Surgery, West China Hospital, Sichuan University, 37 Guoxue Xiang St., Chengdu, Sichuan China

**Keywords:** Endocarditis, Three dimesional transesophageal echocardiography, Tricuspid valve prosthesis, Tricuspid regurgitation, Systemic lupus erythematosus

## Abstract

Libman-Sacks endocarditis (LSE), characterized by verrucous vegetations formation, is a typical cardiac manifestation of autoimmune diseases such as systemic lupus erythematosus (SLE) and antiphospholipid syndrome (APS). It primarily leads to lesions of cardiac valves and mostly involved valves are mitral and aortic, but isolated tricuspid valve involvement is exceptional. Here we reported a 20-years-old female with past SLE history suffered from acute right heart failure caused by multiple tricuspid vegetations and valve regurgitation. The patient recovered following tricuspid valve replacement with a bioprosthesis. Transesophageal echocardiography(TEE), especially real time 3-dimensional (RT3D) TEE provide a better imaging modality for assessing cardiac valvular involvement of LSE. For patients with active SLE/APS course, uncontrolled systemic inflammation may made it difficult for surgical exposure and suture. The durability of bioprosthesis for this patient and the prosthesis selection for tricuspid LSE both need further follow-up and more clinical investigation.

## Background

In 1924, Libman and Sacks originally reported 4 systemic lupus erythematosus (SLE) cases with verrucose vegetative endocarditis [1], and that was the first introduction of Libman-Sacks endocarditis(LSE). Nowadays, LSE have been seen as a typical cardiac manifestation of autoimmune diseases such as SLE and antiphospholipid syndrome(APS). The pathologic changes of LSE involve the formation of fibrin-platelet thrombi on the altered valve, the organization of which leads to valve fibrosis, edema, diffuse thickening, mild inflammatory changes, valve distortion, scarring, and subsequent valvular dysfunction such like stenosis or regurgitation [2, 3]. LSE often involve left heart valves, tricuspid lesions were very rare. And most LSE can be treated with medicine therapy while very few need surgical treatment. Here we reported a rare tricuspid LSE patient with SLE history which underwent open-heart surgery with bioprosthesis replacement.

## Case presentation

A 20-year-old young Asian female presented to our department for continuous weakness and short of breath for more than 2 months on June 2014. The girl’s past history was significant for systemic lupus erythematosus (SLE) more than 3 years but she denied regular oral hormone therapy for nearly 2 years. Symptoms of paroxysmal knee joint ache, paroxysmal nocturnal dyspnea and orthopnea are manifested. She had experienced increased dyspnea on exertion with activities of daily living and increasing lower extremity edema.

**Fig. 1:**
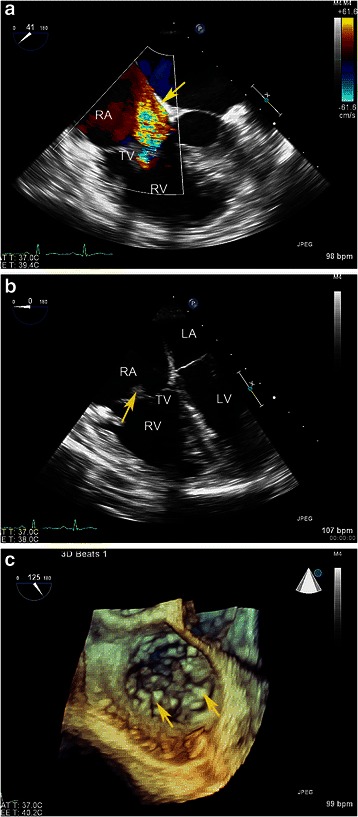
Transesophgeal echocardiography images of the patient before surgery. **a** Tricuspid regurgitation, Yellow arrowhead: wide and reversed blood flow signals at TV site. **b** A large vegetation formation. Yellow arrowhead: A large vegetation adhere to anterior leaflet of TV. **c** Suspicious multiple vegetations on 3D echo image. Yellow arrowheads: multiple verrucous abnormal nodular projections on the leaflet surface. *RA* right atrium, *RV* right ventricle, *LA* left atrium, *LV* left ventricle, *TV* tricuspid valve

After admission the patient had transitional mild fever with the highest temperature of 37.9 °C. Heart auscultation showed systolic murmur at the 4th intercostal space by the left border of sternum. The transthoracic and transesophageal echocardiography (TTE and TEE) showed severe tricuspid regurgitation and a large single vegetation on the atrial surface of anterior leaflet, which was swinged by blood flow. Real time3-dimensional (RT3D)TEE further detected numerous verrucose nodular thickening on the leaflet’s atrial surface (Fig. 1). The computed tomography scan excluded existence of arteritis. Her laboratory test yielded the following: normal regular blood tests, elevated erythrocyte sedimentation rate and normal C-reactive protein level, positive antinuclear antibody (tite 1:1000), decreased complement C3 and C4 levels(C3, 0.50 g/l; C4, 0.11 g/l), and negative anti-double-stranded DNA antibody, negative anticardiolipin antibody and negative lupus anticoagulant. Hepatic and renal functions were all normal just after admission. Blood culture was taken for 3 times consecutively, but no existence of bacteria was shown. History of unhygienic intravenous injection was denied. Prednisone and hydroxychloroquine were given after admission for 2 weeks but the valve vegetations didn’t disappear according to TEE follow-ups, and the patient did show aggravated clinical symptoms of right heart failure such as loss of appetite, edema of lower extremities, polyserositis, hepatolienomegaly and continuous increasing hepatic function indexes.

**Fig. 2:**
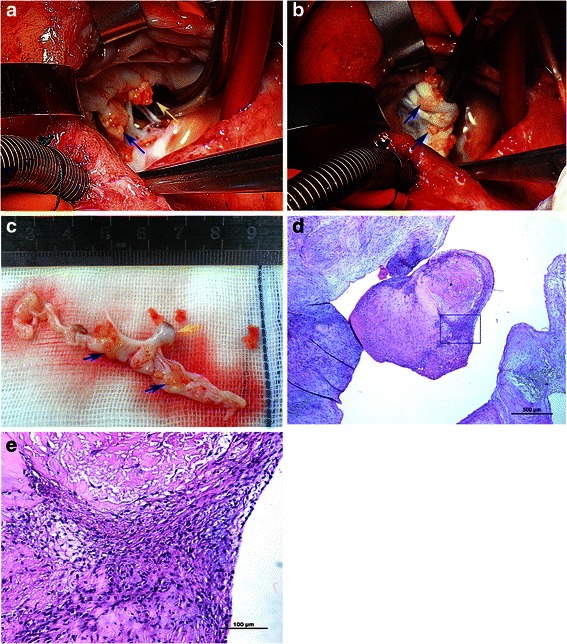
Macroscopy and microscopy of the involved tricuspid valve and vegetation. **a** Yellow arrowhead: The large vegetation, Blue arrowhead: rupture mainchordae tendinae. **b** Blue arrowheads: Multiple verrucous nodular vegetation on the atrial surface of leaflet. **c** Resected tricuspid valve. Blue arrowheads: multiple small vegeatations, Yellow arrowhead: rupture main chordae tendinae. **d** microscopy of the vegetation adhered to the leaflet, Magnification 4×, Hematoxylin and Eosin stain. **e** enlarged square area in (**e**) showing inflammatory cell infiltration and fibrin-platelet thrombi, Magnification 20×, Hematoxylin and Eosin stain

Due to the uncontrolled and evolved right heart failure, the patient received emergent surgery intervention. Considering her uncontrolled SLE and time-limited steroids treatment, the patient was in status of uncontrolled systemic inflammatory response, which leaded to acute pericarditis and pericardial adhesion, also with edema of heart tissue. We had spent more time to expose the heart and establish cardiopulmonary bypass. Intraoperative macroscopy showed a 5 mm*5 mm*5 mm vegetation attached to the apex of tricuspid anterior leaflet, multiple verrucose nodular vegetations tightly adhered to the leaflet and subvavular apparatus. The main chordae tendinae of the leaflet was also ruptured due to vegetation erosion (Fig. 2a–c). Due to the massive vegetations and inflammation-induced tissue weakness, a final decision of valve replacement was made and a 31 mm Medtronic Hancock bioprosthesis was implanted. The surgery and post-operation procedure were both uneventful. The girl presented no symptom of heart failure and discharged 1 weeks later but with continuous follow-ups for heart surgery and further prednisone treatment of SLE. Histopathological examination of the excised vegetation showed inflammation with neutrophil infiltration combined with fibrin-platelet thrombi formation (Fig. 2d–e). The patient was alive 3 months after surgery and echocardiogram follow-up showed normal tricuspid bioprosthesis function without regurgitation.

## Discussion

The verrucous vegetations formation of LSE are seen as a cardiac manifestation of SLE and/or APS. Mitral and aortic valve is the mostly involved valve, while tricuspid valve involvement was rarely reported. Moyssakis reported 38 LSE in 342 SLE patient which were diagnosed by echocardiography, among which there were 24 mitral and 13 aortic involvement. only one tricuspid involvement [4]. Doppler echocardiography can be considered as the diagnostic technique of choice. But sometimes it is very difficult to identify LSE and true infectious endocarditis (IE), for the former may also have fever due to the original immunology diseases and the latter may also have vegetation. Echocardiographically, LSE vegetations appear as masses of varying size and shape with irregular borders and heterogeneous echo-density, which are firmly attached to the leaflet surface and exhibit no independent motion [2]. While the vegetation of IE may typically exhibit independent motion [5]. The role of transesophageal echocardiography (TEE), especially RT3D TEE, had been emphasized in assessing vegetation size in a patient with LSE [6]. And in our case the diagnostic role of RT3D TEE had been also highlighted, which might be more sensitive to the very special verrucous vegetations.

Treatment for LSE consists of drug treatment and surgical intervention. Corticosteroid and anticoagulation drugs are used for LSE drug treatment. Corticosteroids cannot prevent LSE, but they can help healing LSE lesions by lessening inflammation [3, 7, 8]. However they can also increase tissue fibrosis and scarring, finally worsening valvular damage and dysfunction. Nonetheless, appropriate and sufficient steroid therapy to control autoimmune disease activity is important. Anticoagulation therapy is required due to the increased risk of thrombo-embolic events in LSE and current therapeutic guidelines for APS did suggest long-term anticoagulation to prevent thrombo-embolic events [2, 3]. So if the patient with LSE is hemodynamically stable, conservative treatments above should be firstly recommended. Moaref had reported a successfully recovered case of LSE with treatment of prednisolone and hydroxychloroquine, but the patient denied worsening symptoms of heart failure and received drug treatments by months [9]. If with severe intractable symptomatic valvular dysfunction, surgical intervention for LSE may be required [10, 11]. Since our patient got symptoms of acute uncontrolled right heart failure, early surgical intervention even without adequate steroid treatment should be considered.

In the past decade, large studies on the surgical treatment for LSE were mostly focused on patients with mitral LSE. Bouma et al. reviewed the English literatures of surgical treatment for mitral regurgitation caused by LSE and from 1974 to 2010 there were 41 patients with LSE undergoing mitral valve surgery reported in 30 literatures, 13 patients underwent mitral valve repair while 28 underwent replacement [12]. He suggested that for only localized abnormalities with otherwise relatively normal leaflets, repair and rather than replacement was considered as a good surgical opinion. We think the suggestion for mitral site also partly works for tricuspid-involved patient. And we had also reviewed the very few published cases of isolated tricuspid LSE which underwent surgery, and collected types of etiology and surgical procedure of reported cases [13–18] (Table 1). Cases which exhibited vegetations to obstruct valve orifice have the possibility of being treated by vegetation removal and may not need valve replacement. While cases with subvavular apparatus involvement were mainly undergone valve replacement. Wang had recently reported a 40-year-old Asian female with SLE which had similarly multiple vegetations on the subvalvular apparatus and the atrial side of the leaflets of tricuspid valve and a small perforation on a septal leaflet [13]. The patient received sufficient steroid treatment but vegetations still existed and finally underwent mechanical valve replacement due to the difficulty of vegetation removal. We think if subvalvular apparatus were involved, the repair work may be hard and choice of replacement should be considered since the difficulty to reconstruct the subvalvular apparatus (either vegetation removal or repair of chordae tendinae, especially for active SLE/APS patients which suffered total systemic inflammation and tissue edema). In our case we also judged the difficulty of repair due to multiple vegetations formation on the subvalvular apparatus and inflammation-induced tissue weakness which leaded to chordae rupture, finally implanted a Medtronic Hancock porcine bioprosthesis at the tricuspid site.

**Table 1:** Reviews of literature on isolated tricuspid valve surgery for TS/TR caused by Libman-Sacks endocarditis

Reference	Published years	Gender/Age(y)	SLE and/or APS	Adequate steroids usage before surgery	Heart failure before surgery	TS/TR	Morphological changes	Surgical procedure	Follow-up
Laufer et al. [14]	1982	F/9	SLE	Yes	Yes	TR	Dilated annulus and elongated chordae, no vegetations	Mechanical prosthesis replacement	Not mentioned
Ledingham et al. [15]^a^	1988	F/19	SLE		No	TS	a large calcified mass invading the right side of the heart	Bioprosthesis replacement	
						TS	Subsequent tricuspid stenosisafter initial surgery	Removal but no re-implantation of tricuspid prothesis	Alive 5 years post-2^nd^op
Chan-Lam et al. [16]^a^	2001	F/29	APS			TS	two masses that were adherent to the tricuspid valve and intermittently prolapsed through the pulmonary valve.	Surgical removal of the masses	
Falode et al. [17]	2006	F/35	APS	Not mentioned	Yes	TS	massive vegetations involving the tricuspid valve, filling the right atrium	Vegetation removal and valve replacement (type of prothesis not mentioned)	Alive 3 months post-op
Gur et al. [18]	2014	F/20	SLE	Yes	Yes	TS	multiple verrucous vegetations as if being a mass on the anterior and posterior leaflets	Tricuspid valve commisurotomy and Kay annuloplasty	Alive 6 months post-op
Wang et al. [13]	2014	F/40	SLE	Yes	No	TR	several large nodules on the subvalvular apparatus and the atrial side of the leaflets of tricuspid valve; small perforation on a septal leaflet	Mechanical valve replacement	Alive 5 weeks post-op
Bai et al.	2014	F/20	SLE	No	Yes	TR	A large and mutiple tiny vegetations on the atrial side of anterior leaflet with ruptured main chordae tendinae of the leaflet	Bioprothesis replacement	Alive 2 months post-op

*TS* Tricuspid stenosis, *TR* Tricuspid Regurgitation

^a^the full texts of the articles from Ledingham and Chan-Lam ^(15,16)^cannot be acquired through any online database or official websites of the original journals, the author might only get informations from online abstracts

Usually for LSE, it was not very recommended to implant a bioprosthesis since there was reported cases that underwent re-operation in the future, due to rapid calcification, valvulitis and subsequent perforation [19] or massive bioprosthetic thrombosis [20]. So a mechanical prosthesis might provide a comprehensive better result for LSE. But there were no expert suggestion on prosthesis selection for tricuspid site for LSE. For normal consideration, mechanical tricuspid valve replacement (TVR) leads to increased early mortality [21] and occurrence of valve-related events, especially the composite of thrombosis, embolism, and bleeding [22]. Compared to the possibility of mid-to-long-term degeneration and failure of bioprosthesis, we could not tell which kind of prosthesis on tricuspid site should have better outcomes. Considering the prosthesis site and the gender/age of our patient, a mechanical valve may be not very recommended since it might need more intense anticoagulation at tricuspid site which may lead to unexpected embolism-bleeding events and bring harassments for female menstruation and pregnancy. But the durability of bioprosthesis for this patient and the prosthesis selection for tricuspid LSE both need further follow-up and more clinical investigation.

## Conclusion

LSE should be strongly suspected when significant valve vegeation unveiled during the course of SLE and/or APS. Mitral involvement is common but tricuspid LSE is rarely reported. TEE, especially RT3D-TEE, is useful for diagnosis between LSE and IE. Conservative treatment with steroids should be firstly recommended, but patients with untreated and severe intractable symptomatic valvular dysfunction still need surgical intervention. For patients with active SLE/APS course, uncontrolled systemic inflammation may made it difficult for surgical exposure and suture. The prosthesis selection for tricuspid LSE when valve repair is impossible, mechanical or bioprothesis, need further follow-ups and more clinical cases investigation.

## Consent

Written informed consent was obtained from the patient for publication of this case report and any accompanying images. A copy of the written consent is available for review by the Editor-in-Chief of this journal.
